# ‘Trust but verify’ – five approaches to ensure safe medical apps

**DOI:** 10.1186/s12916-015-0451-z

**Published:** 2015-09-25

**Authors:** Paul Wicks, Emil Chiauzzi

**Affiliations:** 155 2nd Street, PatientsLikeMe, Cambridge, MA 02141 USA

**Keywords:** Apps, Asthma, Diabetes, E-health, Insulin, Internet, Mobile health, Smartphone

## Abstract

Mobile health apps are health and wellness programs available on mobile devices such as smartphones or tablets. In three systematic assessments published in *BMC Medicine*, Huckvale and colleagues demonstrate that widely available health apps meant to help patients calculate their appropriate insulin dosage, educate themselves about asthma, or perform other important functions are methodologically weak. Insulin dose calculators lacked user input validation and made inappropriate dose recommendations, with a lack of documentation throughout. Since 2011, asthma apps have become more interactive, but have not improved in quality; peak flow calculators have the same issues as the insulin calculators. A review of the accredited National Health Service Health Apps Library found poor and inconsistent implementation of privacy and security, with 28 % of apps lacking a privacy policy and one even transmitting personally identifying data the policy claimed would be anonymous. Ensuring patient safety might require a new approach, whether that be a consumer education program at one extreme or government regulation at the other. App store owners could ensure transparency of algorithms (whiteboxing), data sharing, and data quality. While a proper balance must be struck between innovation and caution, patient safety must be paramount.

Please see related articles: http://dx.doi.org/10.1186/s12916-015-0444-y, http://www.biomedcentral.com/1741-7015/13/106 and http://www.biomedcentral.com/1741-7015/13/58

## Background

Mobile health apps are software tools that can help users manage their health through a smartphone or tablet, ranging from simple diaries or reminders to more complex programs certified by health authorities as medical devices. The potential for mobile health apps is undoubtedly great, with half a billion users already [1]. However, three linked studies published in *BMC Medicine* question the safety and quality of medical apps in today’s lightly regulated market. Huckvale and colleagues conducted systematic assessments of smartphone apps for calculating insulin dosage [2], educating patients about asthma [3], and the privacy characteristics of “accredited” apps in the National Health Service (NHS) Health Apps Library [4]. Their findings make for sobering reading.

Insulin dose calculation is a basic task, for which we might reasonably assume we can trust a computer better than our human faculties. However, assessment of apps performing this calculation found a litany of errors that force us to consider critically the current ecosystem [2]. It is alarming to read that 91 % of dose calculators lack validation to check the data quality of user input and 67 % risked making an inappropriate dose recommendation. There was a disappointing lack of transparency too, with 70 % lacking documentation for the formula used, 46 % of developers failing to respond to requests for information, and two developers flat-out refusing to share their algorithm with researchers, citing commercial reasons. Quality was no higher for paid apps than free ones, and no higher in the Apple store than the Android store, despite Apple having more stringent entry criteria for apps in general. Most errors pointed patients toward taking a higher dose of insulin than was needed, with the potential for avoidable hypoglycemia.

A review of self-management education apps for asthma, also by Huckvale et al., scored apps on their degree of conformity with evidence-based guidelines in areas such as inhaler technique. The most recent study reviewed apps in 2013 [3] and compared to an earlier review from 2011 [5] found a number of cosmetic upgrades intended to boost user engagement, such as multimedia content, social networking, gamification, sensor integration, and cloud data storage, but sadly with no improvement in the actual quality of content . A third (33 %) lacked correct information about inhaler preparation and mouth positioning, and only half correctly described the proper sequence of inhalation. Peak flow calculators, which provide a measure of how well lungs are functioning, had similar shortcomings to the insulin dose calculators in nine of the apps assessed.

Finally, a third study describes a systematic security and privacy review of apps in the accredited NHS Health Apps Library, a space in which consumers might reasonably assume that such issues would be robustly addressed [4]. However here, too, the authors found inconsistency and poor discipline, with apps storing medical data in ways that left them susceptible to interception or data leaking, as well as highly variable uses of privacy policies. In one case, an app was found to transmit a form of data explicitly claimed not to be transmitted in its privacy policy.

## Health apps are consistently poor in quality

While the authors’ first two studies focused on diabetes and asthma, poor results have been reported in other fields too. For example, a dermatology app claiming to have been downloaded over 35,000 times purported to identify pre-cancerous moles. However, on testing it was found to have just 10 % sensitivity to classify biopsy-proven melanomas correctly [6]. A highly downloaded rheumatology app featuring various calculators was withdrawn for giving users 15–20 % inaccurately higher scores on a disease activity score (DAS28 [7]) for one formula and a 10–15 % lower score than was accurate for another [8].

In order for medical apps to evolve, improved oversight and continuous quality review is required. Centralized oversight by regulatory bodies has the advantage of regulatory expertise and powers to sanction. However, these regulatory bodies are too under-resourced to wade through the sheer volume of apps and there appears to be little appetite to get involved.

Although there are many apps that purport to help with health, in the USA, the Food and Drugs Administration (FDA) will only intervene should an app be found disreputable, and perhaps not even then [9]. Voluntary certification schemes such as the Happtique Health App Certification Program have been piloted [10]. Despite best intentions, however, this program was wound down after only 2 years because a random selection of “certified” apps chosen for review contained major security flaws [11].

In the UK, the NHS Health Apps Library requires developers to complete a structured series of questions about security, quality, and privacy to be reviewed by an internal team. However, in the third linked study by Huckvale et al. [4] the authors found a number of flaws regarding privacy in accredited apps. Moreover, most patients access apps through native stores rather than accredited portals, further highlighting the importance of wariness when it comes to security and privacy issues. In an accredited environment, the speed and revision of review is an important factor when review processes take months or years, while apps can be updated in a matter of weeks and hardware changes regularly [11].

An alternative might be for developers to self-certify according to a checklist in order to reassure the public and maintain their own quality assurance. However, systematic self-certification [12] or a systematic rating scale [13] do not bring more observers to the table but rather rely on the developers’ honesty and competence; there are also few benefits and no sanctions to drive compliance. Peer reviews are offered by journals (e.g., *JMIR mHealth and uHealth*) and commercial sites (e.g., iMedicalApps), but such reviews are few and far between relative to the high number and evolving landscape of health apps. Although important for user satisfaction, rating app quality primarily on dimensions such as visual appeal, functionality, and overall satisfaction without rigorous data quality checking [13] fails to put sufficient emphasis on uncovering the type of data quality issues identified in the studies described here.

## Some proposed approaches to improvement

What would an ideal process look like? Unlike pills or medical devices, there are few centralized gatekeepers between app developers and end-users, no systematic surveillance of harms, and little power for enforcement. Here, we consider five possible approaches that could be taken (Table 1).

**Table 1:** Five potential approaches to improving the quality of medical apps

Approach	Who leads the approach?	Emphasis of approach	Strengths	Weaknesses
Boost app literacy	The medical technology community	Educate consumers on how to make better decision	Empowering, educational, low-cost, no barrier to innovation	Difficult burden remains on patients, no oversight or enforcement
App safety consortium	App developers, safety researchers, regulators, patient advocates	Identify harms arising from health apps	Gathers data, raises concerns appropriately	Low yield, no current infrastructure, funding
Enforced transparency	App Stores and Researchers	Enable external validation by third parties	Continuous quality assessment, enforceable by app stores	Threat to competitiveness, additional work for developers
Active medical review	App Stores	Medical review of every app before release to the public	Robust, enforceable, drives quality and safety	Barrier to innovation, reduces number and diversity of apps, costly, slow
Government regulation	Regulators, e.g., Food and Drugs Administration, Medicines and Healthcare products Regulatory Agency	Medical review of every app before release to the public	Existing powers, enforceable, drives quality and safety	Very slow, cost borne by government, barrier to innovation

### Boost app literacy

The most light-touch approach would be a bottom-up strategy to educate consumers about how to evaluate and interpret their own data in health apps [11]. App developers could voluntarily choose to increase transparency through prominent placement of documentation in the app store that highlights the testing, reliability/validity, data privacy policies, and business model of their medical app. This information could include lay descriptions of the populations(s) on which the app was tested, the context of testing, the validity and reliability of the data collected by the app, and how the app developer will make money or otherwise fund future improvements in the app. Consumers could then place greater faith in what they read if developers have submitted their documentation for independent review and approval. The information provided might also improve a user’s health literacy by highlighting important aspects of the app that they should consider before installation. The challenge to this approach is that even a trained clinician might struggle to access all the relevant literature and systematically assess every version of every app in every permutation of user, much less understand complex security and privacy issues and synthesize them to make a rational decision—most patients could also find this extremely challenging.

### App safety consortium

Given the need for multiple stakeholders to tackle the problem but bearing in mind the view of developers who would resist active control, a second approach would be to convene an app safety consortium of developers, safety researchers, patient advocates, and regulators, which might investigate systems for consumer reporting of adverse events resulting from app use, such as insulin overdose, an approach that has been proposed for patient-reported outcomes in clinical trials of drugs and medical devices [14]. Properly elucidating the level of harm arising from poor app design might draw greater scrutiny, encourage the sharing of best practices, or, while rarely the desired mechanism, encourage litigation that sharpens focus on addressing these issues robustly. Such a consortium would serve as an organizing force to further develop regulatory and risk management frameworks.

### Enforced transparency

A third approach would be for the owners of the app stores to enforce the ability to evaluate medical calculator apps transparently in the same way that ClinicalTrials.gov permits external third parties to look for deviations in protocols, changes in statistical planning, or lack of publication [15] without manually reviewing every trial themselves. In order to access their population of consumers, app developers would be required to submit documentation (viewable by all and accessible through an open database) to be reviewed by anyone but particularly amenable to review by consortia of researchers and clinicians who could evaluate relevant aspects of each app with automated software. This would effectively be “whiteboxing” what was previously a “black box” and allow third parties to develop software that checks the functioning of apps as a service to developers, app store owners, clinicians, and the public. The degree of transparency enforced may require tweaking to ensure the competitive advantage is not eliminated for those developers who have done the hard work to ensure the quality of their product.

### Active medical review

A more active approach would be that those running app stores, such as Apple and Google, take full responsibility for every aspect of security and quality for medical apps as a “benign dictatorship.” After withdrawing all current medical apps in their app stores (which we know from these studies include under-developed programs created by amateurs with no intention of providing ongoing support), they would need to implement a robust testing program staffed by clinicians, security experts, and quality assurance software engineers who would thoroughly vet medical apps before they were released to the public. While this most conservative approach might sound appealing to clinicians and safety enthusiasts, it is also the least likely to succeed.

For instance, Apple already has a complex set of App guidelines in place [16], although as one commentator claims, “the rules are subjective and poorly enforced” [17]. Apps already take a substantial amount of time and energy to review just for basic functionality, let alone the complex verification steps that would be required to remedy some of the issues described by Huckvale and colleagues, and because health apps probably account for a very small proportion of revenue, it would be hard to imagine technology companies taking on the administrative burden as well as the potential for liability should harms arise from apps that have undergone a more rigorous review.

### Government regulation

Any of these approaches is probably still preferable to the final extreme: government regulation of smartphone apps. Only a tiny number of health apps, such as OncoAssist, have gone through European Union Kitemark certification to be qualified as a medical device, a rigorous process that ensures the data they present can be relied upon for clinical decision-making [10]. If the public wanted to be more confident of safety and app store owners did not want to hire a brigade of technologically minded clinicians to review each app, governments could decide to increase the resources available to the existing regulatory bodies to enhance their capabilities and increase the throughput of testing programs. However, this approach likely carries more burden than opportunities.

## Conclusion

Any one of these approaches will add complications and cost to the simple act of downloading an app, but this may still be preferable to avoidable serious adverse events inflicted by software bugs or sloppy practice. Do we want 100,000 medical apps, most of which are shoddy? Or do we want 1,000 that we can rely upon? It is the patients, their caregivers, and their healthcare professionals who should drive what an appropriate level of rigor might be, and ultimately they are the only ones who can exert pressure to change the system. We believe most people would be surprised at the low standards of apps described by these three important studies and disappointed that the safeguards they rely upon in other spheres of life, such as truth in advertising, professional practice standards, or clinical testing of medical products, appear to be absent in this exciting and much-hyped area of techno-utopianism. In considering whether a bottom-up or top-down approach is best, we must also balance innovation and diversity of approaches against patient wellbeing—there is no point “disrupting” the established healthcare system if the new era is not safer for patients than the old one.

As medical innovators, this has been a difficult set of data to fathom. We eagerly look forward to a time when medical apps might be relied upon to do *much* more complex tasks than simply calculate formulae or illustrate inhaler technique; for example, recommending personalized dosage schedules, analyzing patterns in user behavior, interacting with the Internet of Things, perhaps even controlling implanted medical devices. The potential for benefit remains vast and the degree of innovation is inspiring, but it turns out we are much earlier in the maturation phase of medical apps than many of us would have liked to believe. To build the future we want, in which patients can trust their medical apps, we need to verify that they function as intended.
